# Interstitial lung disease in patients with antisynthetase syndrome: a retrospective case series study

**DOI:** 10.1007/s11604-020-01030-3

**Published:** 2020-09-02

**Authors:** Elisa Baratella, Cristina Marrocchio, Rossella Cifaldi, Mario Santagiuliana, Alessandro Marco Bozzato, Paola Crivelli, Barbara Ruaro, Francesco Salton, Marco Confalonieri, Maria Assunta Cova

**Affiliations:** 1 Department of Radiology, University of Trieste, Strada di Fiume 447, 34128 Trieste, Italy; 2 Department of Medicine, Surgery and Health Science, University of Trieste, Strada di Fiume 447, Trieste, Italy; 3 Department of Pneumology, Azienda Sanitaria Universitaria Giuliano Isontina (ASUGI), Strada di Fiume 447, Trieste, Italy; 4 Diagnostic Imaging 2, AOU Sassari, viale S. Pietro 43, Sassari, Italy

**Keywords:** Antisynthetase syndrome, HRCT, Acute interstitial pneumonia

## Abstract

**Purpose:**

Antisynthetase syndrome (ASS) is a rare systemic autoimmune condition associated to the presence of anti-aminoacyl-tRNA synthetase antibodies. Interstitial lung disease (ILD) is the most prevalent manifestation of ASS and is a major determinant of morbidity and mortality. The aim of this study was to describe the radiological characteristics of patients with ASS-associated-ILD in our institution.

**Materials and methods:**

Medical records from 2014 to 2020 were retrospectively reviewed and patients with a diagnosis of ASS and evidence of ILD on HRCT were included. HRCT images were reviewed by two thoracic radiologists in consensus. Five HRCT patterns were defined: cellular non-specific interstitial pneumonia (NSIP), organizing pneumonia (OP), mixed NSIP/OP pattern, acute interstitial pneumonia (AIP) pattern and fibrotic pattern. Descriptive statistics was calculated for all variables.

**Results:**

Twenty-two patients with ASS who met inclusion criteria were included. The disease presented with the typical triad of ASS in 45% of patients, 55% had ILD only at the onset. Cellular NSIP was present in 27% of patients, OP in 23%, mixed NSIP/OP in 9%, AIP in 18% and a fibrotic pattern in 23%.

**Conclusion:**

HRCT findings in ASS-associated ILD are often non-specific; nevertheless, it is important to consider this diagnosis, especially in patients presenting with acute onset of symptoms.

## Introduction

Antisynthetase syndrome (ASS) is a rare systemic autoimmune condition classified among the idiopathic inflammatory myopathies, associated to the presence of anti-aminoacyl-transfer RNA synthetase antibodies. These antibodies are directed against enzymes that attach amino acids to their cognate transfer ribonucleic acid during polypeptide synthesis. At present, eight antisynthetase antibodies have been identified, the most frequent being anti-Jo1 (histidyl), followed by anti-EJ (glycyl), anti-PL7 (threonyl) and anti-PL12 (alanyl) [1]. ASS is characterized by a classical triad of Interstitial Lung Disease (ILD), myositis, and positivity to antisynthetase antibodies and other less specific symptoms such as polyarthritis, mechanic’s hands, Raynaud’s phenomenon, or symptom overlapping with Sjogren syndrome and systemic sclerosis [2].

Interstitial lung disease (ILD) is the most prevalent manifestation of ASS, occurring in 71–100% patients, and may be the only clinical manifestation at the onset of the disease [2, 3]. ILD is a major determinant of morbidity and mortality in antisynthetase syndrome, and the extension of lung involvement correlates with the prognosis of the disease [4]. High-resolution computed tomography (HRCT) plays a main role in ASS in diagnosing ILD, in identifying the pattern of involvement at the onset of symptoms and in assessing the resolution, stability or progression of the pulmonary alterations during follow-up [5–7].

The most common radiological patterns described in ASS-associated ILD are non-specific interstitial pneumonia (NSIP) and organizing pneumonia (OP). Less commonly, usual interstitial pneumonia (UIP) and acute interstitial pneumonia (AIP) have been reported. Nevertheless, the evidence is currently based on retrospective studies or case reports [8–10]. In our clinical practice, we often encountered patients finally diagnosed with ASS who had HRCT findings reported to be less frequent in this syndrome. It is important to be aware of all the possible ASS presentations on HRCT, to consider this syndrome among the differential diagnoses even in atypical cases, avoiding delays in the diagnosis.

In this study we report our experience describing the clinical characteristics and the HRCT radiological findings of a series of patients with a diagnosis of ASS in the last five years at our institution.

## Materials and methods

The study was approved by the local internal ethical review board and has been performed in accordance with the ethical standards laid down in the Declaration of Helsinki. All patients gave informed consent prior to their inclusion in the study. Medical records in our Institution from 2014 to 2020 were retrospectively reviewed and patients were included if they met the following inclusion criteria: (i) 18 years or older; (ii) the diagnosis of antisynthetase syndrome; (iii) HRCT performed since the diagnosis with evidence of ILD; (iv) no other diseases presenting with abnormal findings on chest CT (e.g. lung cancer, pulmonary infections, sarcoidosis). Patients were diagnosed with ASS syndrome according to the criteria proposed by Solomon et al. [11], after multidisciplinary consultation involving at least a thoracic radiologist, an expert rheumatologist, a pneumologist and a pathologist. Demographics and relevant clinical data including disease presentation and symptoms at diagnosis were registered. HRCT was performed with 256-row multidetector CT system (Brilliance iCT 256, Philips, Best, The Netherlands) and acquired during single breath hold at full inspiration, with the patient in a supine position. Technical parameters were as follows: rotation time, 270 ms; beam collimation, 128 × 2 × 0.625 mm; normalized pitch, 0.975; *z*-axis coverage, 160 mm; reconstruction interval, 0.3 mm; section reconstruction thickness, 1 mm; tube voltage, 120 kV; tube current (effective mA), 280–400 depending on patient size; and field of view, 40 cm. CT images were analyzed at standard lung window settings (window level of − 600 HU and window width of 2000 HU) and mediastinal window setting (window level 400–500 HU and window width 20–40 HU).

Images were reviewed in consensus by two thoracic radiologists with 15 and 10 years of experience, who were aware that only patients with ASS were included but were blinded to clinical symptoms and other patients’ characteristics. Discrepant interpretations were resolved by consensus through the involvement of a third reader with 20 years of experience.

The following CT findings were evaluated: reticulations, traction bronchiectasis, honeycombing, ground glass opacities (GGO), air-space consolidations. According to the Fleischner society recommendation, reticulations were inter- and intra-lobular septal thickening; traction bronchiectasis was defined as bronchial and bronchiolar irregular dilatation caused by fibrotic changes of the surrounding lung parenchyma; honeycombing presented as clustered cystic air spaces with thick and well-defined walls, usually in dorsal and subpleural regions of the chest; GGO was defined as a hazy increased lung attenuation that does not obscure the bronchovascular structures; air-space consolidation was a homogeneous increase in pulmonary parenchymal attenuation [12].

Based on CT findings, five HRCT patterns were defined: cellular non-specific interstitial pneumonia (NSIP), organizing pneumonia (OP), mixed NSIP/OP pattern, acute interstitial pneumonia pattern and fibrotic pattern [5]. According to the ATS/ERS classification of idiopathic interstitial pneumonia, cellular NSIP was characterized by GGO generally bilateral, symmetrical and with a prevalently medial and basal distribution [5]. Fibrotic NSIP, characterized by irregular septal thickening and signs of fibrosis such as traction bronchiectasis and architectural distortion, was included in the fibrotic pattern. OP presented with air-space consolidations in a typically subpleural and peribronchial distribution and with migratory behavior. The pattern was considered mixed NSIP/OP when consolidations were superimposed on a background of GGO [13]. In case of acute presentation, within a month, acute interstitial pneumonia (AIP pattern) was reported if bilateral patchy GGO were present, often associated with areas of consolidation. When signs of lung fibrosis (honeycombing, traction bronchiectasis, irregular septal thickening and reticulations) were present, the pattern was classified as fibrotic. In this category, the definite and probable UIP and the fibrotic NSIP patterns were included. UIP was reported according to the ATS/ERS/JRS/ALAT guidelines as definite, when honeycombing and reticular opacities were present in a basal and peripheral distribution, often with traction bronchiectasis, and as probable when the honeycombing was missing [5]. The CT information was completed by all patients' clinical characteristics.

Other signs of chest involvement were noted, including pleural effusion, pericardial effusion, and presence of pulmonary hypertension. Pulmonary hypertension was reported when the main pulmonary artery had a diameter of 29 mm or more or the ratio of the diameter of the main pulmonary artery to the diameter of the ascending aorta was greater than 1:1. Indirect signs of pulmonary hypertension were considered to be presence of right heart disease, such as right ventricular enlargement and hypertrophy, or suggestive parenchymal features, including mosaic attenuation.

Descriptive statistics was calculated for all variables. Results are expressed as mean ± SD; frequencies and proportions are provided for categorical variables.

## Results

Twenty-two patients with ASS who met inclusion criteria were included. Sixteen patients were female (73%) and six were males (27%) with a mean age at diagnosis of 52 ± 6 years. Ten patients (45%) presented with the typical triad of ILD, myositis, and serum positivity for anti-aminoacyl-tRNA synthetase antibodies, whereas the remaining patients (12/22, 55%) had only ILD as clinical manifestation at diagnosis. Signs and symptoms of pulmonary involvement were dyspnea in 19 (86%) and cough in 17 patients (77%); four patients (18%) presented with acute respiratory failure. At physical examination we observed skin signs, i.e. erythema, in only one patient.

Regarding antisynthetase antibodies, anti-Jo-1 was detected in six patients, anti-OJ in one, anti-PL-12 in one, anti-PL-7 in one and anti-EJ in one patient at admission. Antibodies of collagen vascular diseases were also evaluated; in particular, anti-SSA were present in three patients, anti-SSB in one patient, antinuclear antibodies (ANF) were present in five patients, and anti-RF were present in one patient i.e. anti-neutrophil cytoplasmic antibodies. No other autoantibodies were observed. An increased serum level of myogenic enzyme was observed in 4 patients.

**Fig. 1:**
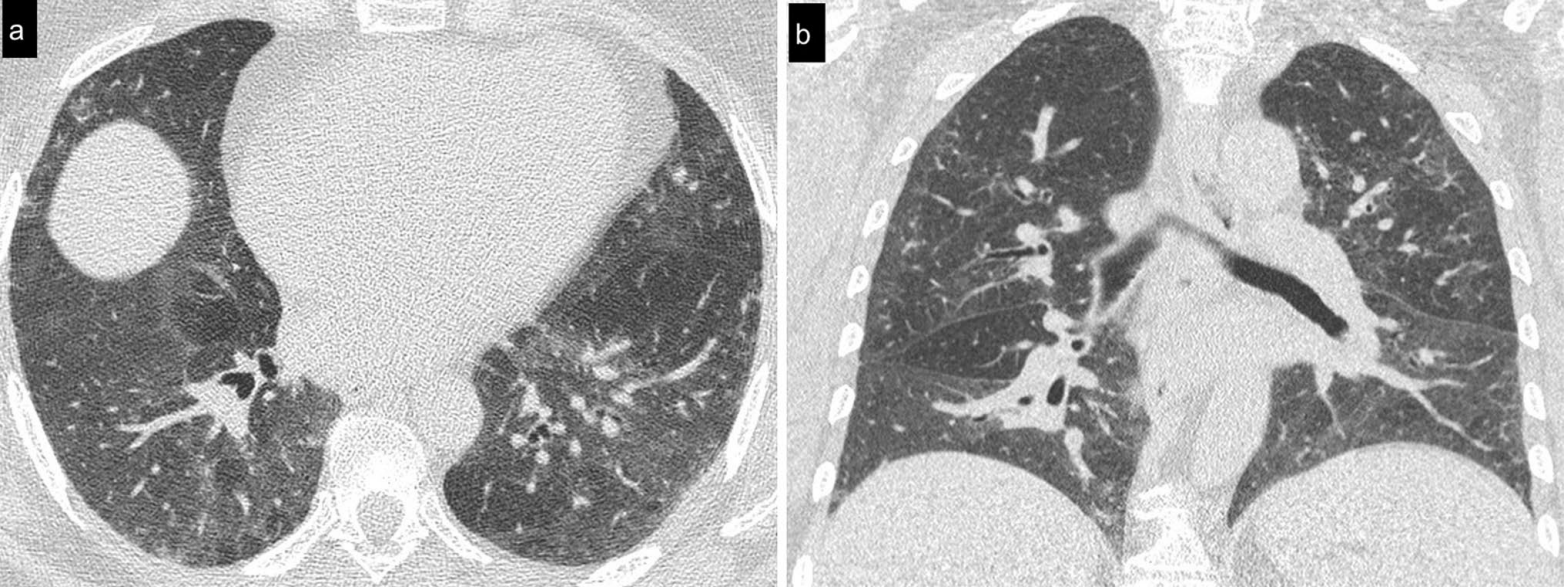
Cellular NSIP pattern: the multiplanar axial (**a**) and coronal (**b**) reconstructions show diffuse areas of ground glass opacities without traction bronchiectasis

**Fig. 2:**
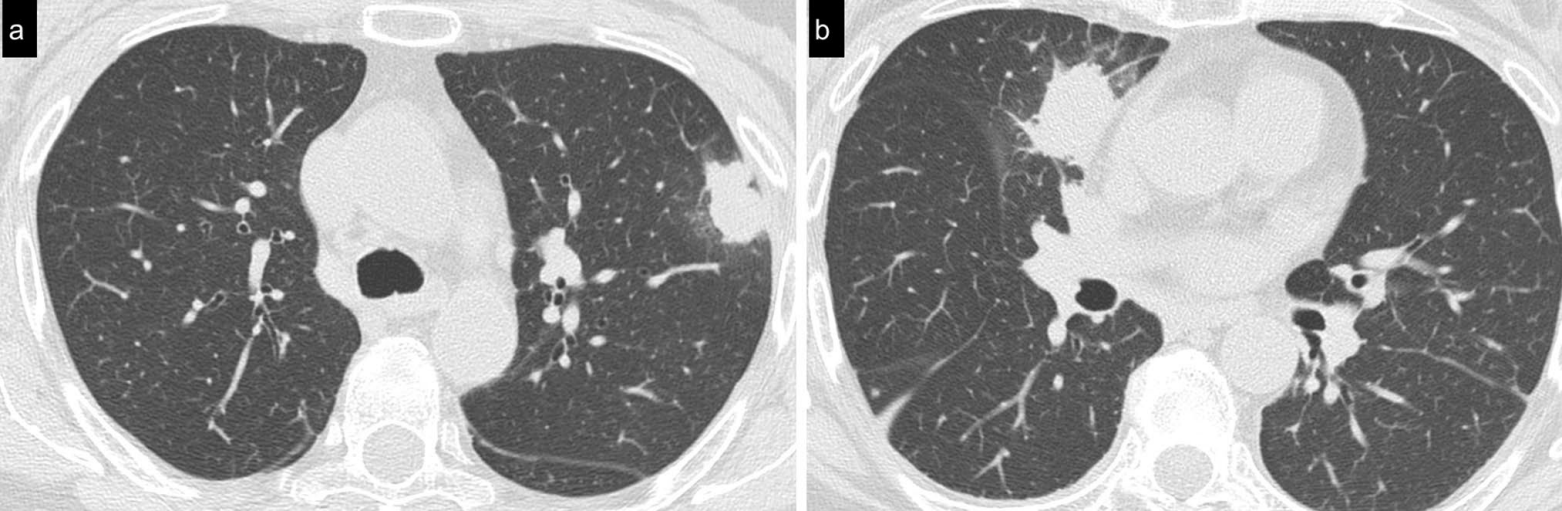
Organizing pneumonia pattern: air-space consolidations surrounded by a ground glass halo were present bilaterally

**Fig. 3:**
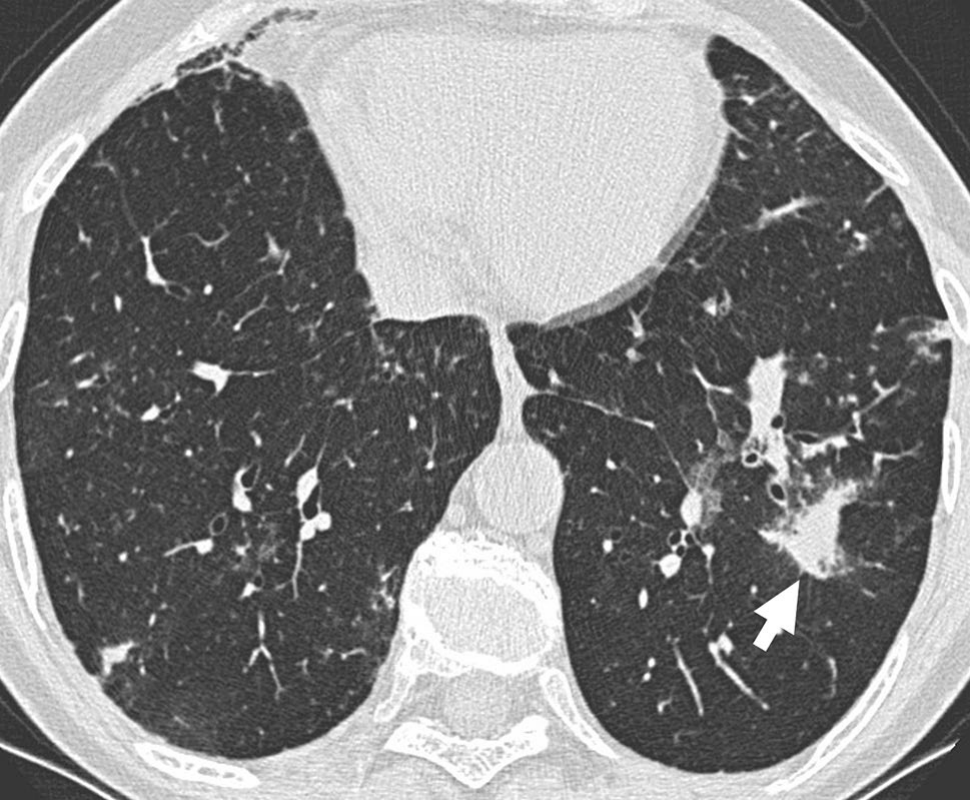
Organizing pneumonia pattern: air-space consolidation with a peribronchial distribution (arrow) is present in the lower left lobe

**Fig. 4:**
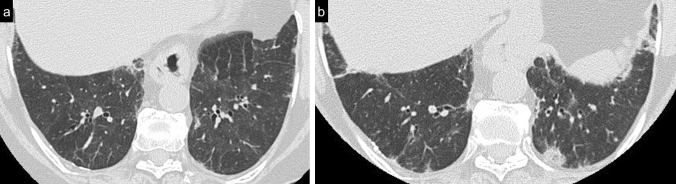
Mixed NSIP/OP pattern: axial CT images of the same patient at different levels show consolidations superimposed on a background of ground glass opacities

**Fig. 5:**
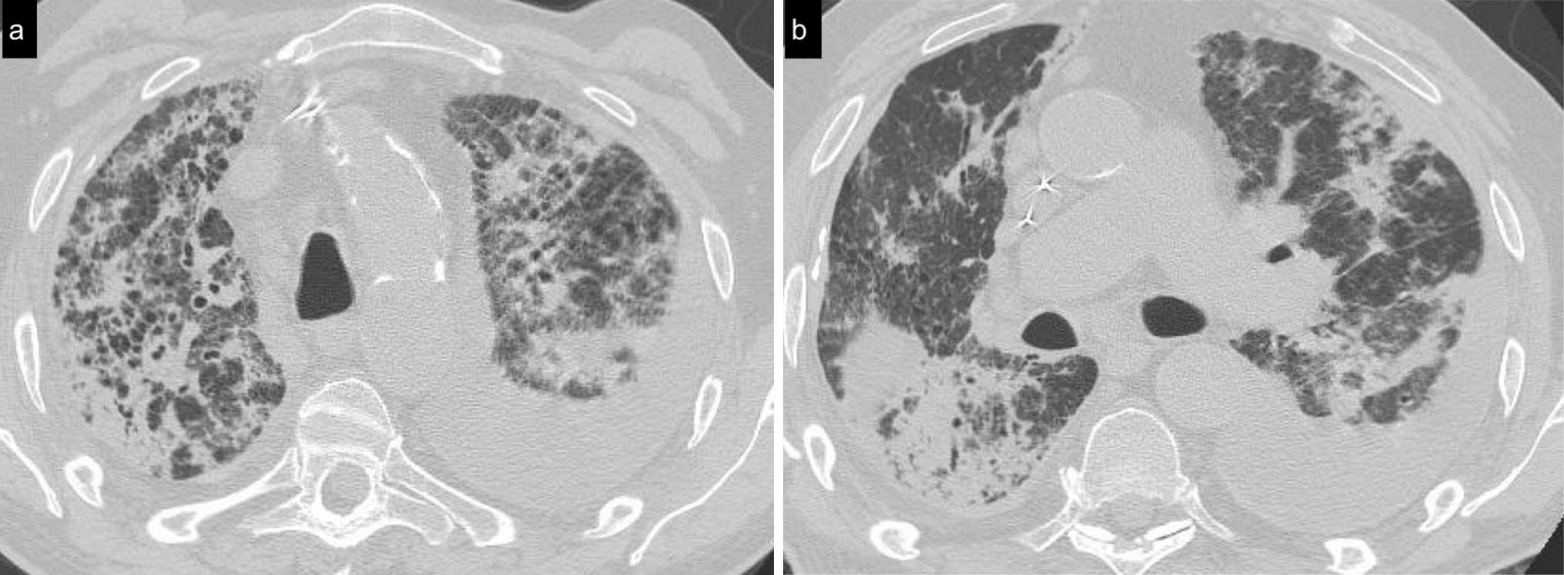
Acute interstitial pneumonia pattern: bilateral inhomogeneous patchy ground glass opacities with areas of consolidation, non-dependent areas of sparing and some traction bronchiectasis are present

**Fig. 6:**
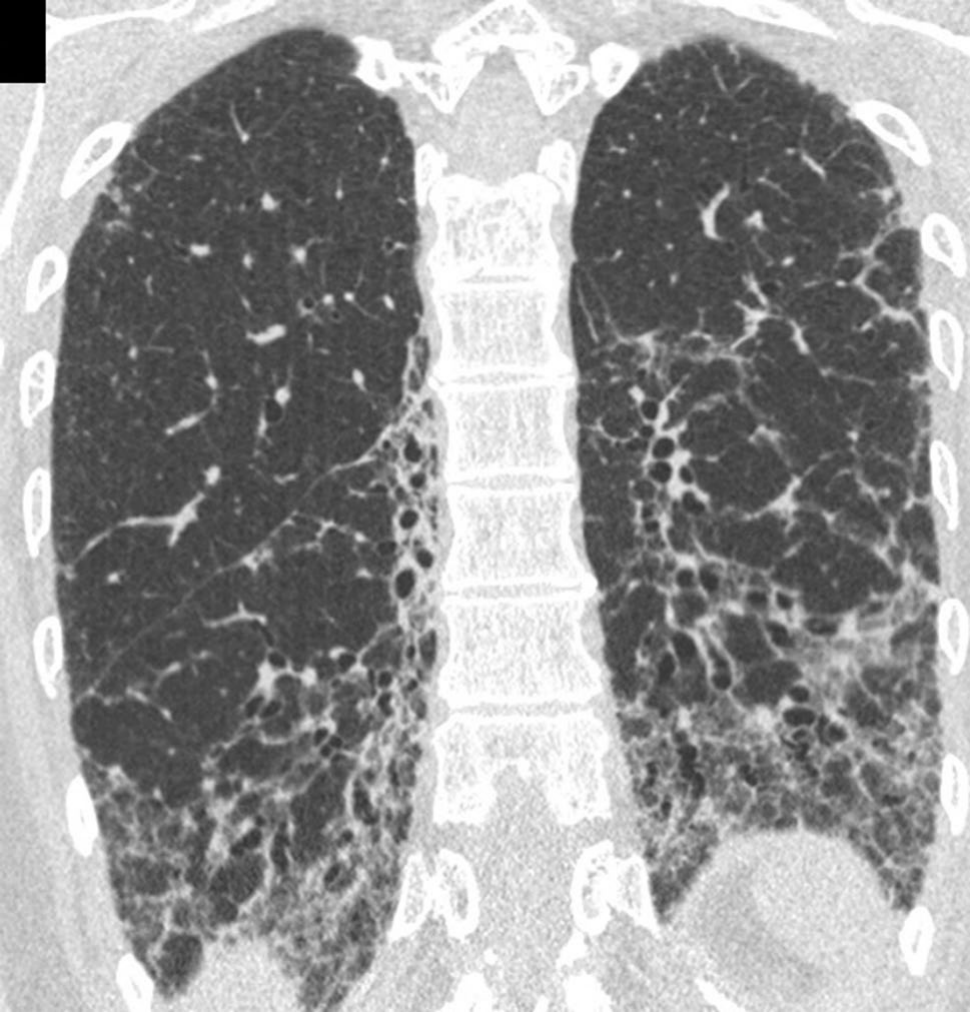
Fibrotic NSIP pattern. The multiplanar coronal reconstruction of this CT scan demonstrates extensive bilateral ground glass opacities with traction bronchiectasis with a peripheral and basal distribution

**Fig. 7:**
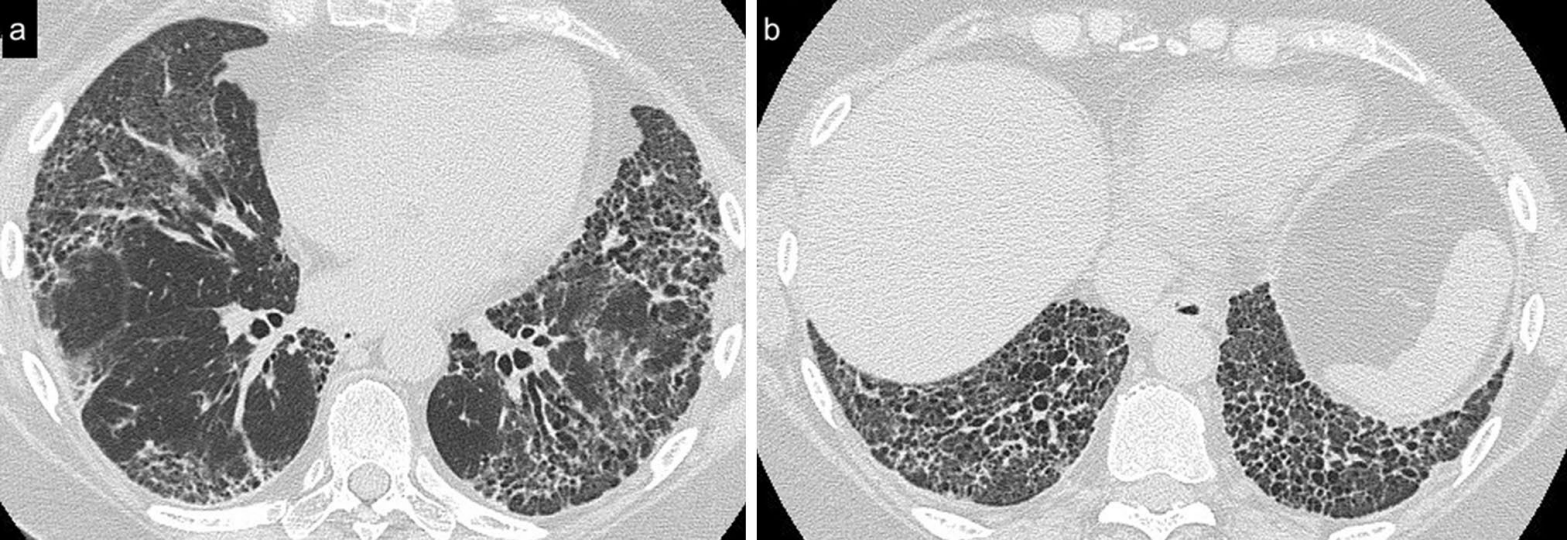
Typical usual interstitial pneumonia (UIP) pattern. Multiplanar axial reconstructions at different levels show an irregular inter- and intra-lobular septal thickening, with a subpleural, basal predominance, traction bronchiectasis and bronchiolectasis (**a**) and honeycombing (**b**), which is clearly seen in the basal region

**Fig. 8:**
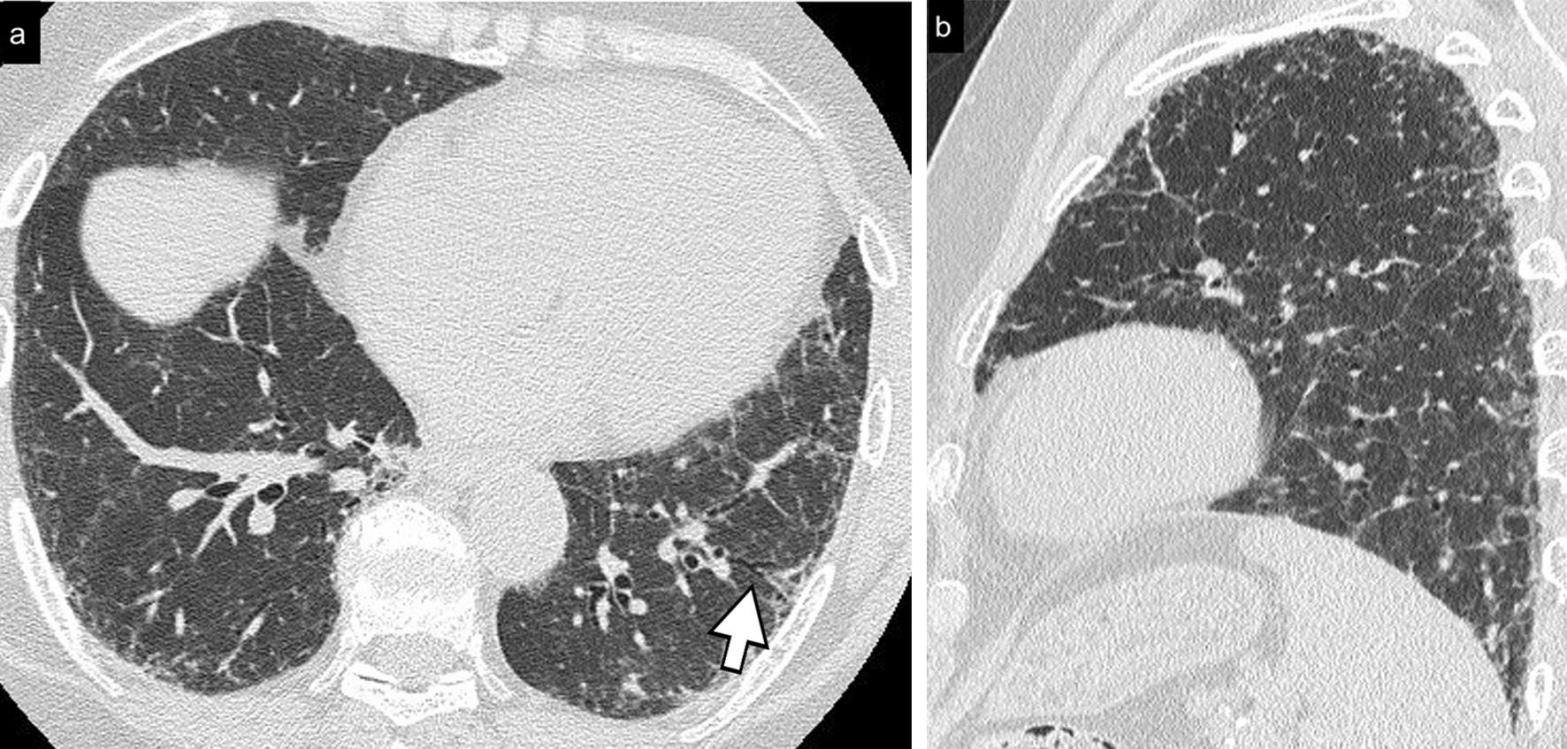
Probable UIP pattern, axial (**a**) and sagittal (**b**) multiplanar reconstructions: irregular inter- and intra-lobular septal thickening, with a subpleural, basal predominance. Traction bronchiectasis and bronchiolectasis are seen in the fibrotic areas (arrow); irregular thickening of the pleural surface is better visualized in the sagittal plane. Honeycombing is not present

Cellular NSIP or OP pattern were the presenting patterns in 50% of patients, specifically 6 (27%) had cellular NSIP (Fig. 1), 5 (23%) OP (Figs. 2, 3), and 2 (9%) had a mixed NSIP/OP pattern (Fig. 4). The HRCT of the 4 patients (18%) who presented with acute respiratory failure showed an AIP pattern, with extensive and inhomogeneous ground glass opacities, areas of consolidations and sparing of non-dependent regions of the lungs (Fig. 5). Fibrotic changes were present in 5 patients (23%) with honeycombing, traction bronchiectasis and reticular opacities (Figs. 6, 7, 8). Specifically, 2 patients were diagnosed with definite UIP, 1 patient with probable UIP and 2 patients with fibrotic NSIP.

**Fig. 9:**
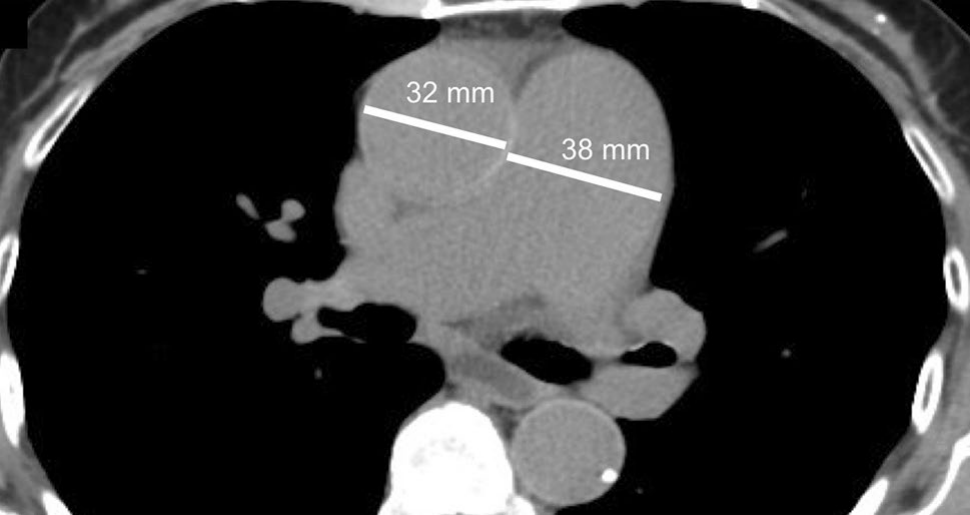
Pulmonary hypertension: the main pulmonary arterial diameter measured at the level of its bifurcation on the axial CT scan is 38 mm and the ratio of the diameter of the main pulmonary artery to the diameter of the ascending aorta is greater than 1:1

Pleural effusion was present in 9 of the twenty-two patients (41%) and pulmonary hypertension in 6 cases (27%) (Fig. 9). Pericardial effusion was a less common finding, occurring in only 3 patients.

## Discussion

The present study reports the clinical and radiological characteristics of a series of twenty-two patients with a diagnosis of antisynthetase syndrome and evidence of interstitial lung disease on HRCT.

ASS is a rare systemic autoimmune disease diagnosed in the presence of antisynthetase antibodies and compatible clinical features, in particular ILD and/or inflammatory myositis. Because of heterogeneity in the observed phenotype and severity of the disease, diagnosis of ASS remains difficult. The clinical and demographic findings in our patients were in line with those reported in ASS patients. The disease was more prevalent in middle-aged women and in 45% of cases it presented with the classic triad of ILD, myositis and presence of antisynthetase antibodies. In 55% of cases, ILD was the only clinical presentation at the onset of the disease, and this underlines the importance of HRCT evaluation and correct interpretation in these patients [2, 14]. Current evidence on HRCT patterns in ASS-associated ILD is based predominantly on retrospective case series and case reports. NSIP has been reported as the most common pattern on HRCT in patients with ILD secondary to autoimmune etiology, in particular in patients with antisynthetase syndrome, followed by OP. Less common is the occurrence of usual interstitial pneumonia [8–10]. In our case series, only half of the patients presented with cellular NSIP and OP patterns. Fibrotic changes in the lung parenchyma were found with the same prevalence as OP. In previous reports, the UIP pattern has been reported to be associated with a worse prognosis [15].

An interesting finding of this study is that ASS had an acute presentation with acute interstitial pneumonia in 4 of 22 patients, accounting for almost 20% of cases. Acute respiratory failure is an uncommon presentation of ASS and may be an unsuspected cause of ARDS, especially if no other extra-pulmonary symptoms of the disease are present. The typical HRCT finding in these patients is an acute interstitial pneumonia pattern, with bilateral patchy GGO often associated to areas of consolidations, and this was the presentation in all our patients with acute onset. Identifying the cause of the acute respiratory failure is a crucial step for initiating a targeted treatment and improving diagnosis, therefore ASS should be considered among the possible causes and, in cases in which it is suspected, immunological tests should be included in the diagnostic work-up [16].

Chest findings in antisynthetase syndrome are not limited to the lung parenchyma and the other manifestations of the disease should always be noted, especially pulmonary hypertension. Although pleural effusion is quite a common finding in patients with connective tissue diseases, in particular in rheumatoid arthritis and systemic lupus erythematosus, it is not frequent in patients with ASS [17]. Only few cases have been reported of ASS-associated pericardial effusion, and indeed this finding was present in only 3 cases in our experience [18].

The radiological characteristics of ASS are often non-specific. The differential diagnosis is broad and has to be made with other main causes of ILD, both idiopathic and secondary, including collagen vascular diseases, drug toxicity, hypersensitivity pneumonitis and asbestosis [19]. To reach the final diagnosis, a multidisciplinary evaluation is highly recommended to evaluate the clinical, serological and radiological findings in each patient [6, 20].

Our study has some limitations. This is a case series with a relatively small sample size, which is a limitation inherent to the rarity of the disease. Moreover, due to its retrospective nature, patients had heterogeneous disease characteristics and not uniform timing of HRCT examinations. Nevertheless, about 50% of our patients presented with findings reported to be less common in ASS. This should improve the awareness of the wide spectrum of HRCT findings in ASS to consider this disease in the differential diagnosis also in challenging cases and especially in those patients presenting with an acute onset and no extra-pulmonary manifestations of the disease.

## Conclusion

HRCT patterns in ASS-associated ILD are often non-specific, but it is important to maintain a high suspicion especially in those patients presenting with acute respiratory failure.
